# SplitAx: A novel method to assess the function of engineered nucleases

**DOI:** 10.1371/journal.pone.0171698

**Published:** 2017-02-17

**Authors:** Richard A. Axton, Sharmin S. Haideri, Martha Lopez-Yrigoyen, Helen A. Taylor, Lesley M. Forrester

**Affiliations:** MRC Centre for Regenerative Medicine, SCRM Building, The University of Edinburgh, Edinburgh bioQuarter, 5 Little France Drive, Edinburgh, United Kingdom; Temple University School of Medicine, UNITED STATES

## Abstract

Engineered nucleases have been used to generate knockout or reporter cell lines and a range of animal models for human disease. These new technologies also hold great promise for therapeutic genome editing. Current methods to evaluate the activity of these nucleases are time consuming, require extensive optimization and are hampered by readouts with low signals and high background. We have developed a simple and easy to perform method (SplitAx) that largely addresses these issues and provides a readout of nuclease activity. The assay involves splitting the N-terminal (amino acid 1–158) coding region of GFP and an out-of-frame of C-terminal region with a nuclease binding site sequence. Following exposure to the test nuclease, cutting and repair by error prone non-homologous end joining (NHEJ) restores the reading frame resulting in the production of a full length fluorescent GFP protein. Fluorescence can also be restored by complementation between the N-terminal and C-terminal coding sequences in trans. We demonstrate successful use of the SplitAx assay to assess the function of zinc finger nucleases, CRISPR hCAS9 and TALENS. We also test the activity of multiple gRNAs in CRISPR/hCas9/D10A systems. The zinc finger nucleases and guide RNAs that showed functional activity in the SplitAx assay were then used successfully to target the endogenous *AAVS1*, *SOX6* and *Cfms* loci. This simple method can be applied to other unrelated proteins such as ZsGreen1 and provides a test system that does not require complex optimization.

## Introduction

Zinc finger nucleases (ZFN), Clustered Regularly Interspersed Short Palindromic Repeats (CRISPR) and Transcription Activator-Like Effectors (TALEs) are powerful tools which can be used for genome editing[1–6]. To avoid delivering ineffective editing tools to cells and live animals, it is imperative to test their functionality prior to delivery. There are many different methods that have been developed to assess whether engineered nucleases are functional. These include surveyor mutation detection, gene replacement and single strand annealing assays[7–9]. These methods can require extensive optimisation, are time consuming and suffer problems of high background noise [10]. We have developed a novel reporter assay (SplitAx) that can be used to assess the efficacy of genome editing tools including ZFN, CRISPR/Cas9/D10A systems, and TALENs. It is based on the property that GFP may be split into two fragments. GFP consists of 11 anti-parallel β strands. The GFP protein can tolerate the addition of internal peptide sequence at specific locations between antiparallel β strands 4 and 5, 7 and 8, as well as 8 and 9 [11]. We exploited this property introducing a genome editing binding site between antiparallel β strands 7 and 8 so that a stop mutation prevents read through into the C-terminus of GFP preventing fluorescence. We demonstrate that the addition of ZFNs, TALENs and hCAS9 with guide RNAs followed by NHEJ leads to restoration of the GFP fluorescence. This method is robust, requires little optimisation and has the potential to be developed for high throughput screening of nucleases. Here, we show that the SplitAx assay is functional with GFP and an unrelated fluorescent coral protein, ZsGreen1. Sequencing of break points after exposure to the genome editing system illustrates that following error prone NHEJ there are two mechanisms by which the assay functions: by frameshift restoring an open reading frame and through complementation with the N-terminus and C-terminus of the fluorescent protein.

## Materials and methods

### Design and cloning of SplitAx vectors

The AAVS1-SplitAx-GFP, AAVS1-SplitAx-ZsGreen1, SOX6-SplitAx-GFP and Cfms-SplitAx-ZsGreen1 were generated as a double stranded DNA oligos (http://eu.idtdna.com/site) so that the binding site would be out of frame preventing read through into 3’end of the coding sequence. 50ng of each double stranded oligo were incubated at 72°C with dNTP and Emerald Taq polymerase (Clontech) to add adenine bases for TA cloning (Life Science). Colonies were selected and grown, plasmid DNA extracted and verified by DNA sequencing. The correctly sequenced clones were sub cloned by EcoRI digest into a cut EcoRI pCAG-ASIP-ires-Puro vector to generate the completed SplitAx vector. Alternatively, immediately upstream and downstream of the genome binding site are the restriction enzymes sites NotI and XhoI. These allow an alternative method to rapidly exchange the genome editing binding sites into the pCAG-ASIP-ires-Puro (SplitAx Vector).

### Transfection protocols

All transfections were performed with Xfect (Takira Clontech), on 200,000 293FT cells plated in 6 well dishes. 500ng of the AAVS1-GFP-SplitAx or AAVS1-ZsGreen1-SplitAx were co-transfected with 1000ng of AAVS1 Zinc Fingers (P662L and P622R) [12, 13]. 1000ng of CRISPR gRNA_AAVS1-T1, CRISPR gRNA_AAVS1-T2 (Addgene Church George)[14], AAVS1 TALENs Left and Right (Addgene Zhang)[15], gRNA-SOX6 1a, 1b, 2a or 2b, gRNA-Cfms 8a, 8b or 9a were co-transfected with their respective 500ng GFP-SplitAx or ZsGreen1-SplitAx and 1000ng hCAS9 or D10A nickase. The cells were cultured for 44–48 hours and then analysed by flow cytometry (BD LSR Fortessa) and with FlowJo data analysis Software. All data shown are the result of three independent experiments and in each experiment a parallel well was transfected with a pCAG-GFP vector to assess transfection efficiency. Transfection efficiencies of over 80% were routinely observed.

### Genomic targeting of the *AAVS1*, *SOX6* and *Cfms* loci

For targeting the *AAVS1* locus 10 million hiPSCs (SFCi55) were electroporated (BioRad 320V, 250uF) with 40ug of the targeting vector AAVS1 Promoter KLF1 mCherry reporter construct plasmid and 5ug of each AAVS1 ZFN P662L and P662R plasmids. For targeting the SOX6 locus 10 million hiPSCs (SFCi55) were electroporated (BioRad 320V 250uF) with 40ug of the SOX6 targeting plasmid, 5ug hCAS9 plasmid and 5ug of the gRNA_SOX6-1a. For targeting the *Cfms* locus 10 million murine ES cells (E14) were electroporated (Biorad 320V 250uF) with 40ug of the *Cfms* targeting vector plasmid and 10ug D10A nickase with 4ug of gRNA_SOX6-8a and 4ug of gRNA_SOX6-9b.

### Screening targeted clones

Puromycin selection (0.4ug/ml) of the *AAVS1* targeted cells resulted in resistant colonies. These were picked expanded prior to genomic DNA isolation and PCR screening to identify correctly targeted clones using primers A4 and A5 for the 5’ screen. To screen correctly targeted clones at the 3’ end primers A1, A2 and A3 were used (S1 Table).

The SOX6 targeting vector electroporated cells were sorted for mOrange at 48 hours post electroporation. The cells were plated at low density and grown till colonies formed. Colonies that had retained the mOrange were expanded prior to genomic DNA isolation. PCR amplification with primers P1, P2 and P3 identified correctly targeted clones (S1 Table).

Selection of the *Cfms* targeted clones with G418 (300 μg/ml). Clones were expanded prior to genomic DNA isolation and PCR screening. PCR amplification with primers C1, C2 and C3 identified correctly targeted clones (S1 Table).

### Genomic DNA isolation and PCR screening

DNA was isolated (Bioscience KIT). 100ng of genomic DNA was used in each PCR reaction with Hot Start Emerald Taq polymerase (Clontech-Takara) with the relevant primers (S1 Table). The cycling conditions were as follows: 94°C for 2 minutes, and 34 cycles of 94°C for 15 seconds, 55°C for 30 seconds and 72°C for 1 minute 20 seconds, and then 1 cycle at 72°C for 10 minutes.

### Construction of targeting vectors

The KLF1 promoter mCherry reporter construct was generated as follows: Firstly the mCherry reporter was PCR cloned with the primers mCherry for and mCherry_rev (S1 Table) into the vector PL452 using the restriction enzymes KpnI and EcoRI. The KLF1 promoter region including an intronic enhancer [16] was amplified by PCR from genomic DNA with primers KLF1a_for and KLF1a_rev (S1 Table). The KLF1 promoter region was then cloned into PL452 following KpnI restriction enzyme ligation cloning. The βGlobin-Poly A was added by PCR cloning using primers βGlobin PA_for/rev followed by EcoRI restriction enzyme ligation cloning downstream of the mCherry reporter. The KLF1 promoter-mCherry-PolyA cassette was then amplified by PCR using the primers KLF1PZPuro_for and PZdage_rev (S1 Table) and cloned by Age1 restriction digest ligation into an AgeI restriction enzyme cut PZDONOR AAVS1 Puro vector (Sigma Aldrich).

The SOX6 C-terminal targeting vector was generated by PCR cloning 5’ and 3’ homology arms from genomic DNA using primer pairs S1, S2, S3 and S4, and cloned by restriction enzyme/ligation cloning NotI 5’ Homology arm and XhoI 3’ homology arm, respectively into the destination vector pBluescript KS2+ (S1 Table). The T2A-BFP-PolyA cassette was synthesises as a double stranded oligo (IDT) and cloned into the 5’Homology and 3’Homology arm pBlueScript KS2+ vector using restriction BamHI. The LoxP-EF1 alpha promoter was amplified by PCR with primers E1 and E2 (S1 Table) and cloned with EcoRI whilst mOrange-PolyA-LoxP was synthesised as a double stranded oligo (IDT) and then cloned into pBlueScript KS2+-5’-3’-T2aBFP-PolyA-EF1 alpha with the restriction enzymes ClaI.

The C-terminal Cfms targeting vector was generated by PCR cloning 5’ and 3’ homology C4, C5 and C6 and C7, respectively) arms into the vector PL452 using KpnI and NotI restriction enzymes respectively. The MMP12-PolyA cassette was generated as two large double stranded oligo fragments IDT. These were joined by restriction enzyme ligation (HindIII) and subsequently cloned into the destination vector PL452 containing the 5’ and 3’ Cfms homology arms using the restriction enzyme ApaI.

## Results

### Functional testing with the SplitAx reporter assay

**Fig 1:**
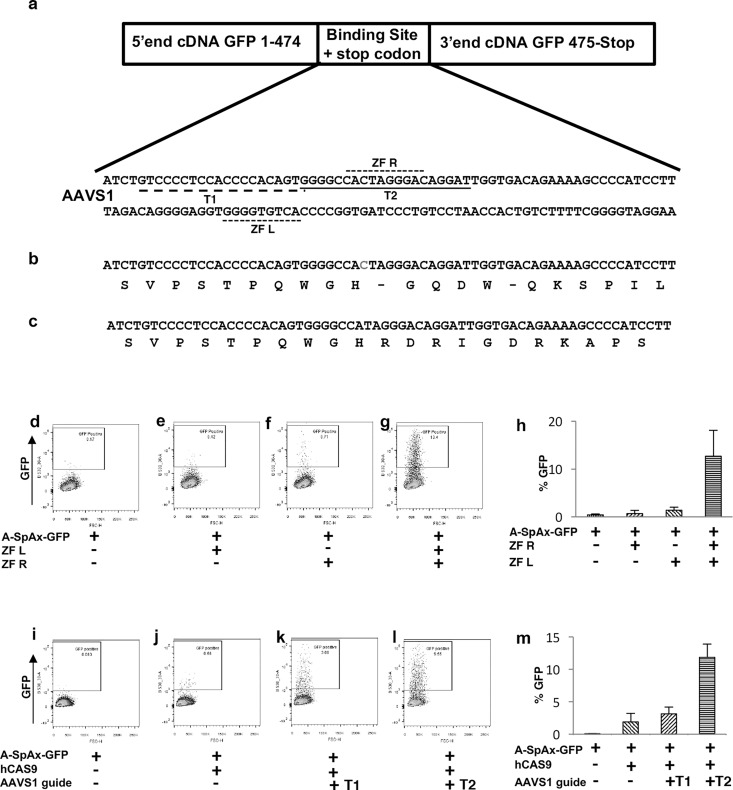
Functional validation of the GFP-AAVS1 SplitAx reporter assay with zinc fingers and CRISPR/CAS9 system. Schematic of the GFP cDNA with the N-terminus and C-terminus separated by the *AAVS1* binding site. The DNA sequence of the *AAVS1* binding site is shown and the location of zinc finger left (ZF L), Zinc finger right (ZF R), *AAVS1* guide RNAs T1 and T2 underlined (a). The translated DNA sequence of the *AAVS1* binding site with stop codons (-) (b). The translated DNA after genome editing. In this case a 1 bp deletion removes the stop codons and allows in frame of translation of the C-terminal GFP resulting in fluorescence (c). Representative flow cytometry plots of 293FT cells 44–48 hours after transfection with GFP-AAVS1 SplitAx only (d), GFP-AAVS1 SplitAx with single AAVS1 Zinc Finger Left (Zn L) (e), GFP-AAVS1 SplitAx with AAVS1 single Zinc Finger Right (Zn R) (f), and GFP-AAVS1 SplitAx with both AAVS1 Zinc Finger Lefand/Zinc Finger Right (Zn L, Zn R) (g). Quantification of flow cytometry data for the GFP-AAVS1 SplitAx with the AAVS1 Zinc Fingers (+), cells not transfected with a plasmid (-). Data shown as mean +/- SD (n = 3) (h). Representative flow cytometry plots of 293FT cells 44–48 hours after transfection with GFP-AAVS1 SplitAx only (i) GFP-AAVS1 SplitAx and hCAS9 (j), GFP-AAVS1 SplitAx, hCAS9 CRISPR and gRNA_AAVS1-T1 (k), GFP-AAVS1 SplitAx, hCAS9 CRISPR and gRNA_AAVS1-T2 (l). Quantification of flow cytometry data for the GFP-AAVS1 SplitAx with the CRIPSR gRNA_AAVS1- T1 or T2 and hCAS9 (+), cells not transfected with a plasmid (-). Data shown as +STDev (n = 3) (m).

We developed a novel assay, termed SplitAx, to screen for genome editing function using the fluorescent reporter GFP sequence split into two regions at a specific location (S1 Fig). For assessing *AAVS1* locus targeting the SplitAX vector consisted of sequence encoding the 5’ end of the GFP cDNA (1-474bp), an intervening sequence specific to the *AAVS1* locus followed by sequence encoding the 3’ end of the GFP cDNA (Fig 1A and S1 Fig). The *AAVS1* specific sequence was designed so that it introduced stop codons upstream of the 3’ cDNA end of GFP preventing read through (Fig 1B and S1 Fig). Following the addition of a zinc finger endonuclease and repair by error prone NHEJ, indel mutations are introduced. In the example shown, a 1bp deletion restores the reading frame that results in the translation of a full length GFP protein (Fig 1C and S1 Fig).

Transfection of 293FT cells with the AAVS1-GFP-SplitAx vector alone or with a single ZFN (ZF) resulted in no/low fluorescent signal (Fig 1D–1F). Transfection with the AAVS1-GFP-SplitAx vector and a pair of *AAVS1*-specific zinc finger nucleases resulted in cutting and repair by error prone NHEJ restoring the fluorescent GFP signal (Fig 1G). This occurs by the introduction of indels into the sequence causing frame shift mutations that restore the reading frame of the C-terminal GFP. To demonstrate reproducibility three separate experiments were carried out and are represented in the graph (Fig 1H).

These zinc finger nucleases were also able to target the endogenous *AAVS1* locus in a human induced pluripotent stem cell (iPSC) line (SFCi55) with a KLF1 reporter construct. Puromycin resistant clones were expanded and screened by PCR. 95% (20 of 21) clones were targeted correctly (S2 Fig).

The *AAVS1* SplitAx vector was then tested with hCAS9 and guide RNAs specific to the *AAVS1* locus. Transfection of 293FT cells with the AAVS1-GFPSplitAx vector and hCAS9 or AAVS1-GFP-SplitAx vector resulted in no or low fluorescent signals (Fig 1I and 1J). Transfection with the AAVS1-GFP-SplitAx vector, hCAS9 and gRNA_AAVS1-T1 or T2(14) resulted in a fluorescent GFP signal (Fig 1K and 1L). Comparison of the two different guide RNAs T1 and T2 in the SplitAx assay concurred with published data showing that T1 cuts less efficiently than T2(14). The experiments were performed three times to demonstrate reproducibility and these are represented graphically (Fig 1M).

We also validated a pair of TALENs specific for the *AAVS1* locus (15) (Addgene) and demonstrate that these also are functional with the AAVS1-SplitAx assay (S3 Fig).

**Fig 2:**
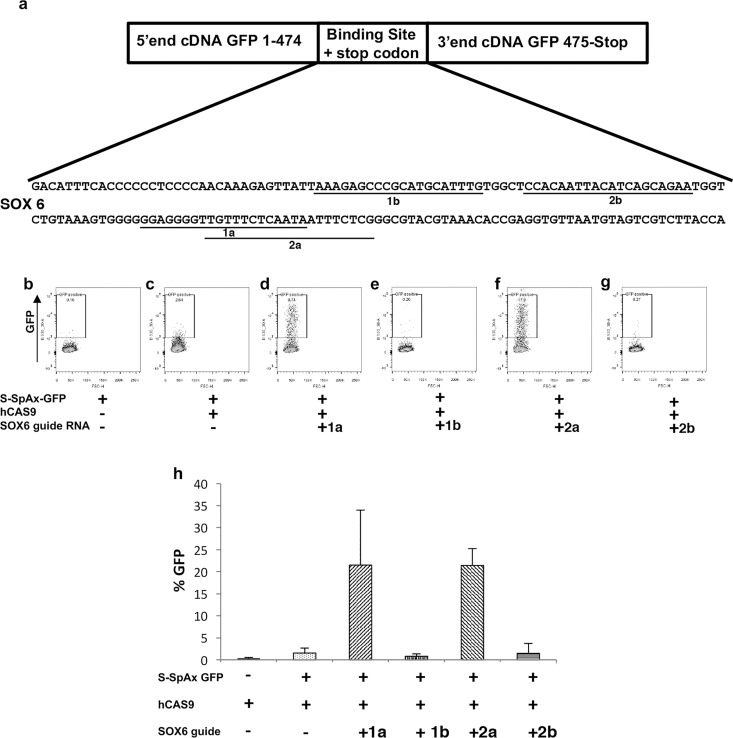
Functional validation of the GFP-SOX6-SplitAx reporter assay with *SOX6* gRNAs and hCAS9. Schematic diagram of the 5’ and 3’end of GFP separated by the SOX6 binding site. The DNA sequence of the SOX6 binding site is shown and the location of the gRNA_SOX6-1a, 2a, 1b and 2b are underlined (a). Representative flow cytometry plots of 293FT cells 44–48 hours after transfection with GFP-SOX6 SplitAx only (b), GFP-SOX6 SplitAx with hCAS9 (c), GFP-SOX6 SplitAx, hCAS9 with gRNA_SOX6-1a (d), GFP-SOX6 SplitAx, hCAS9 with gRNA_SOX6-1b (e), GFP-SOX6 SplitAx, hCAS9 with gRNA_SOX6-2a (f) and GFP-SOX6 SplitAx, hCAS9 with gRNA_SOX6-2b (g). Quantification of flow cytometry data for the GFP-SOX6 SplitAx and hCAS9 with the gRNAs_SOX6 (+), cells not transfected with a plasmid (-). Data shown is mean +/- SD (n = 3) (h).

To test the SplitAx system using another genetic locus, we generated a SOX6-GFP-SplitAx vector (Fig 2A). Transfection with the SOX6-SplitAx vector alone or in the presence of hCAS9 resulted in low fluorescent signal (Fig 2B and 2C). Transfection with the SOX6-SplitAX vector, hCAS9 and gRNA_SOX6-1a, gRNA_SOX6-1b, gRNA_SOX6-2a or gRNA_SOX6-2b resulted in either high or low fluorescent activity (Fig 2D–2G). The experiments were repeated in triplicate to demonstrate reproducibility and are represented graphically (Fig 2H).

*SOX6* guide RNAs, selected based on their activity in the SplitAx assay were then used to target the genomic locus in hiPSCs. The targeting efficiency was 5% (2 positive clones out of a total of 40), (S4 Fig).

**Fig 3:**
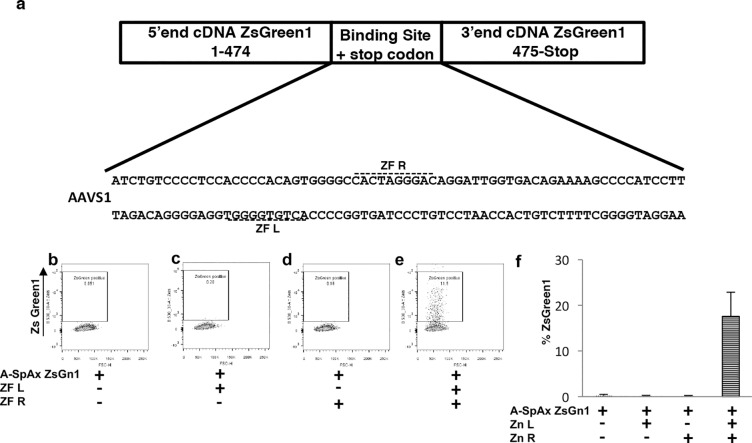
Functional validation of the ZsGreen1-AAVS1 SplitAx reporter assay with AAVS1 zinc fingers. Schematic of the ZsGreen1 cDNA with the N-terminus and C-terminus separated by the AAVS1 binding site. The DNA sequence of the AAVS1 binding site is shown and the location of zinc finger left (ZF L), Zinc finger right (ZF R) (a). Representative flow cytometry plots of 293FT cells 44–48 hours after transfection with ZsGreen1-AAVS1 SplitAx only (b), ZsGreen1-AAVS1 SplitAx with AAVS1 Zinc Finger Left (Zn L) (c), ZsGreen1-AAVS1 SplitAx with AAVS1 Zinc Finger Right (Zn R) (d), and ZsGreen1-AAVS1 SplitAx with AAVS1 Zinc Finger Left/Zinc Finger Right (Zn L, Zn R) (e). Graphical representation of flow cytometry data for the ZsGreen1-AAVS1 SplitAx with the AAVS1 Zinc Fingers (+), cells not transfected with a plasmid (-). Data shown as mean +/- SD (n = 3) (f).

We then tested whether the SplitAx assay could be developed using a different fluorescent protein. We elected to test the coral protein (ZsGreen1). The amino acid conservation between GFP and ZsGreen1 is 26% (S5 Fig). It was noted that there was amino acid conservation at residues KQ which is the critical residue at which to introduce the nuclease binding site at position 158 (S5 Fig). We developed an AAVS1 ZsGreen1-SplitAx vector consisting of the sequence 5’ end of the ZsGreen1 cDNA (1-474bp) and immediately incorporated the sequence specific to the *AAVS1* locus downstream, followed by 3’ cDNA sequence encoding ZsGreen1 (475bp–end). As before, the *AAVS1* locus was designed so that it introduced a stop codon upstream of the C-terminal ZsGreen1 preventing read through (Fig 3A).

Transfection of 293FT cells with the AAVS1-ZsGreen1-SplitAx vector alone and co-transfection with AAVS1-ZsGreen1-SplitAx and single ZFN Left (L) or Right (R) demonstrated low fluorescence (Fig 3B–3D). Co-transfection of AAVS1-ZsGreen1-SplitAx and AAVS1 Zinc finger 662L and 662R resulted in a strong fluorescent signal (Fig 3E). The experiment was performed in triplicate (n = 3) to verify reproducibility and is represented graphically (Fig 3F).

**Fig 4:**
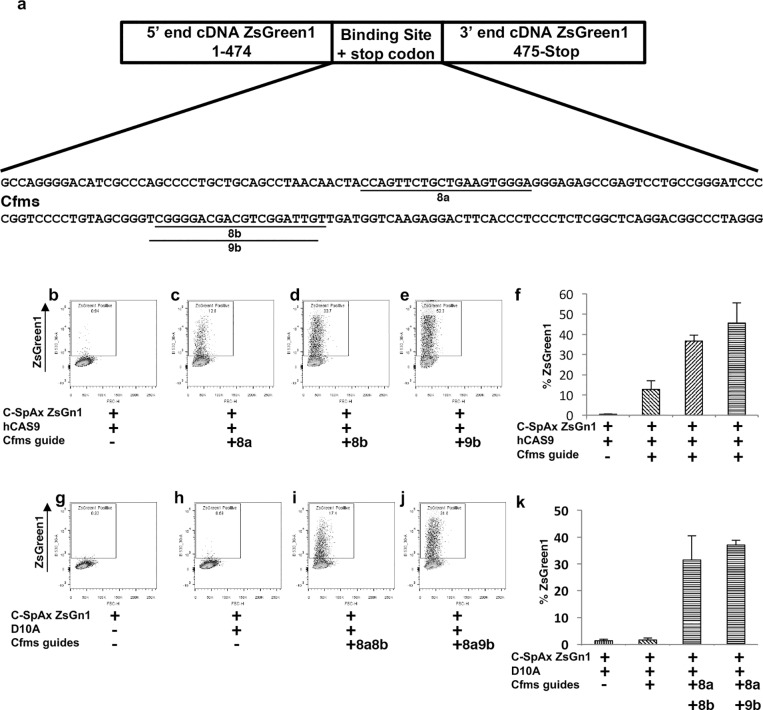
Functional validation of the ZsGreen1-Cfms-SplitAx reporter assay with Cfms gRNAs and hCAS9 or D10A nickase. Schematic diagram of the 5’ and 3’end of Zs Green1 separated by the *Cfms* binding site. The DNA sequence of the Cfms binding site is shown and the location of the gRNA_Cfms-8a, 8b and 9b are underlined (a). Representative flow cytometry plots of 293FT cells 44–48 hours after transfection with ZsGreen1-Cfms-SplitAx with hCAS9 (b), ZsGreen1-Cfms-SplitAx, hCAS9 with gRNA_Cfms-8a (c), ZsGreen1-Cfms-SplitAx, hCAS9 with gRNA_Cfms-8b (d), ZsGreen1-Cfms-SplitAx, hCAS9 with gRNA_Cfms-9b (e). Quantification of flow cytometry data for the ZsGreen1-Cfms- SplitAx and hCAS9 with the gRNAs_Cfms (+), cells not transfected with a plasmid (-). Data shown as mean +/- SD (n = 3) (f). Representative flow cytometry plots of 293FT cells 44–48 hours after transfection with ZsGreen1-Cfms-SplitAx only (g), ZsGreen1-Cfms-SplitAx with D10A nickase (h), ZsGreen1-Cfms-SplitAx, D10A nickase with gRNA_Cfms-8a and8b (i), ZsGreen1-Cfms-SplitAx, D10A nickase with gRNA_Cfms-8a and 8b (j). Graphical representation of flow cytometry data for the ZsGreen1-Cfms- SplitAx and D10A nickase with the gRNAs_Cfms (+), cells not transfected with a plasmid (-). Data shown as mean +/- SD (n = 3) (k).

A *Cfms* targeting binding site was also engineered into the ZsGreen1 SplitAx vector (Fig 4A). The Cfms-ZsGreen1-SplitAx vector with hCAS9 showed virtually no fluorescent signal (Fig 4B). The addition of gRNA_Cfms-8a, gRNA_Cfms-8b and gRNA_Cfms-9b resulted in a strong fluorescent signal (Fig 4C–4E). Three independent experiments were performed and are represented in the graph (Fig 4F). We also demonstrated that the Cfms-ZsGreen1-SplitAx vector can be used with the D10a nickase enzyme. The Cfms-ZsGreen1-SplitAx vector alone or with the D10A nickase enzyme gave virtually no fluorescent signal (Fig 4G and 4H). The addition of RNA gRNA_Cfms-8a and 8b resulted in a high fluorescent signal (Fig 4I). Similarly, the addition of gRNA_Cfms8a and 9b also resulted in a high fluorescent signal (Fig 4J). Three independent experiments were performed and are represented in the graph (Fig 4K).

We then demonstrated that *Cfms* guide gRNAs that were selected based on the SplitAx assay, gRNA_Cfms-8a and gRNA_Cfms-8b were successfully used to target the genomic locus in mouse ESCs using the D10A nickase system. The targeting efficiency was 6% (3 positive clones out of a total of 50)(S6 Fig).

**Fig 5:**
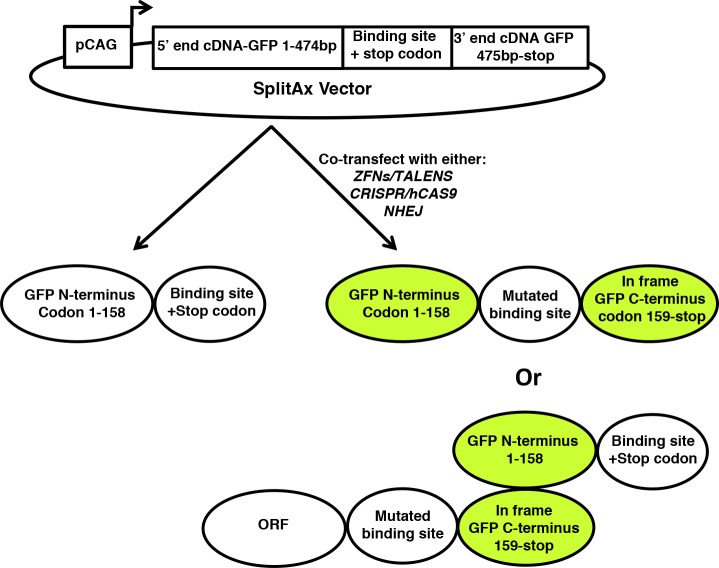
Schematic diagram illustrating the different mechanisms of how the SplitAX assay functions. The vector consisting of the pCAG promoter, the GFP cDNA (N-terminus 1-474bp), a genome editing binding site containing a stop codon which is out of frame with the GFP cDNA C-terminus (475-end). In the absence of exposure to a specific genome editing tool, the full length GFP protein is not expressed. Exposure of the GFP-SplitAx to a genome editing tool creates a double strand break. Repair by non-homologous end joining (NHEJ) mutates the binding site restoring the open reading frame (ORF) of GFP resulting in fluorescence. The second mechanism involves the repair of the double strand break by NHEJ resulting in an N-terminal ORF in frame with the C-terminal GFP. The C-terminal GFP can complement with the N-terminal GFP expressed from a different vector leading to restored fluorescent activity.

Sequencing of the AAVS1-GFP-SplitAx vector after it had been cut with the AAVS1 zinc fingers p662L and P662R and repaired by NHEJ revealed the mechanisms by which the SplitAx system functions. In one sequence the *AAVS1* binding site had been mutated by deleting 10 bp (Δ10) resulting with the N-terminus of GFP in frame with the C-terminus of GFP leading to restored fluorescent activity (Fig 5 and S7 Fig). Three sequences contained the following mutations Δ79+53, Δ91 +110 and Δ93+83, respectively (S7 Fig). The effect of these mutations was to remove the stop codon generating an Open Reading Frame containing the C-terminal GFP protein. The C-terminal GFP can interact with the N-terminal GFP through complementation [17] (Fig 5).

## Discussion

A number of technologies have been developed to screen for the functional activity of genome editing systems including the surveyor mutation detection, episomal gene repair, traffic light reporter or homology dependent GFP repair assays have been developed to screen for the functional activity of genome editing [7, 8, 18–21]. These systems can require extensive optimisation and cannot be monitored in real time. The novel SplitAx assay described here is easy to perform, can be monitored in real time and does not require extensive optimisation.

The basic mechanistic principle of the SplitAx system is that GFP consists of 11 antiparallel β strands. GFP can tolerate additional protein sequence at amino acid position 158 (between antiparallel β strands 7 and 8) and this addition does not affect its fluorescent activity [11, 22]. The SplitAx vector consists of sequence encoding the N-terminus (amino acid 1–158) and C-terminus (amino acid 159-end) separated by an engineered genome editing binding site. The C-terminal sequence of GFP is out of frame with the N-terminal sequence and so a full length fluorescent protein cannot be generated. Following exposure to an engineered nuclease (e.g. ZFNs, TALENs or hCAS9) system and the subsequent repair by NHEJ results in the introduction of frameshift mutations and restoration of the reading frame of GFP and the production of a fluorescent protein.

Using this biosensor assay, we designed SplitAx vectors for three genetic loci, *AAVS1*, *SOX6* and *Cfms*, and have validated these with a variety of genome editing tools including ZFNs, CRISPR/CAS9/D10A and TALENs. The SplitAx assay can be used to quickly monitor whether genome editing tools are functional. Importantly, we have shown that AAVS1 zinc finger nucleases, SOX6 guide RNAs and Cfms guide RNAs that were selected based on their activity in the SplitAx assay, were capable of mediating targeting events at endogenous loci in human and mouse PSCs. This assay could potentially be used to confirm that lack of endonuclease activity of mutant CAS9 proteins that have been designed for alternative function such as gene activation.

Transfection of the SplitAx vector and genome editing tools predictably introduce double strand breaks which are repaired by error prone non homologous end joining (NHEJ) resulting in deletions or insertions of DNA [23, 24]. We hypothesised that a change in the frame shift in the *AAVS1* sequence of -1, -4 or +1 or +4 or any triplet combination/deletion/insertion of this will restore the open reading frame with the C-terminal GFP fragment and generate a fluorescent signal within the cell (Fig 5A). In addition we have shown that the SplitAx assay can also function through an alternative mechanism of complementation (Fig 5B). We have tested 3 different binding sites in the context of a SplitAx vector. The binding sites range in size from 67bp- 89bp. It may be possible to incorporate larger or smaller binding sites into the SplitAx vector. As a matter of prudence, it would be important to consider when designing the binding region whether there is a start codon with a putative Kozak signal [25]. The consequence of this might be expression of C-terminus of the fluorescent protein and complementation with the N-terminus resulting in fluorescent activity in the absence of exposure to a genome editing tool.

Other assays have described introducing a stop codon in the genome editing binding site near the 5’ end of the GFP gene or linking multiple fluorescent proteins in different reading frames [21, 26]. Double strand breaks followed by NHEJ can lead to frameshift mutations and have a 1 in 3 chance that the GFP protein will restore the open reading frame and yield a fluorescent signal. Since there are two mechanisms that can give rise to the fluorescent reporter signal in the SplitAx assay, the sensitivity of this assay is greater than those that rely on a Cis acting frameshift alone.

We have demonstrated that the fluorescent protein ZsGreen1 can be split with a genome editing binding site in a similar manner to GFP. The ZsGreen1 SplitAx vector produces a higher signal and lower signal to noise ratio when compared to GFP, owing to its brighter spectral properties [27]. Since other fluorescent proteins share this property of splitting the N-terminus and C-terminus, it may be possible to test the functional activity of genome editing tools intended for one step mutations in multiple genes simultaneously.

The genome editing tools that showed high functional activity in the SplitAx assay were used successfully to target endogenous loci in hiPSCs (*AAVS1* and *SOX6*) or mouse ESCs (*Cfms*) demonstrating that the plasmid-based SplitAx assay is able to identify editing tools that can function at the genomic level where other factors, including epigenetic factors could affect their activity. However this study did not test tools that were non-functional in the SplitAx for their ability to function at the genome level and so we cannot state that there is a direct correlation between the two systems. Nevertheless, given that the SplitAx requires little optimisation and is easy to perform we believe it provides an attractive alternative to established techniques for the selection of functional tools. The SplitAx assay could also be developed as a high throughput format to screen for functional nucleases or as a method to evaluate off targeting by engineering SplitAx vectors with predicted off target sites. Finally, the SplitAx vector has been designed with the restriction enzymes NotI and Xho1 to facilitate the rapid exchange of the genome editing binding sites.

## Supporting information

S1 Fig DNA and translated protein sequences of GFP and AAVS1-GFP-SplitAx vector. (a)DNA and translated protein sequences of GFP. Amino acid Q158 is marked in red. This is the position in which the genome editing binding site is inserted to split the GFP-N-terminus from the GFP-C-terminus. (b)DNA sequence of AAVS1-GFP-SplitAx vector.Amino acid Position 158 is marked in red, followed by Not1 restriction sites (underlined). AAVS1 genome editing binding site (shaded yellow) followed by Xho1 restriction sites (broken line). (c)DNA and translated protein sequence of AAVS1-GFP-SplitAx vector. Amino acid position 158 is shown in red and stop codons shown as dashes (-). (d)DNA and translated protein sequence of AAVS1-GFP-SplitAx following genome editing. Following a 1 bp deletion at nucleotide position 519 the complete GFP open reading frame is restored.
(DOCX)

S2 Fig Targeting of the AAVS1 locus with the KLF1 promoter mCherry reporter. (a) Schematic diagram of the AAVS1-KLF1-mCherry reporter vector used to target the *AAVS1* locus with zinc fingers P622L and P622R. Left Homology Arm, Splice Acceptor/2A peptide, Puromycin selectable cassette (P), Poly A (PA), KLF1 Promoter, mCherry reporter followed by the *AAVS1* Right Homology Arm (not to scale). (b) Schematic illustration of the *AAVS1* locus, endogenous promoter, exon 1 and the target site between the Left Homology, Right Homology Arm and exon 2. (c) Targeted *AAVS1* locus with the KLF1 reporter vector. Arrows indicate primers used to screen 5’ and 3’ end of the targeting site and solid bars indicate the PCR amplicons. Screen for targeted events using 5’ primers A4 and A5 and 3’ primers are A1, A2 and A3. (d) PCR products from 5’ PCR using primers A4 and A5. Clone 9 indicates that it is targeted at the 5’ end. (e) PCR products from 3’ PCR using internal vector primers and an external primer. Clone 3 indicates a random targeting event whilst clone 9 indicates a targeted event into the AAVS1 locus. WT is genomic DNA from untreated iPS cells and 0 is a negative PCR control. (f) Sequencing trace clone 9 of the 3’ external PCR showing that this PCR amplicon is specific to the *AAVS1* locus.
(DOCX)

S3 Fig Functional validation of the GFP-AAVS1 SplitAx reporter assay with AAVS1 TALENs. (a) Schematic of the GFP cDNA with the N-terminus and C-terminus separated by the AAVS1 binding site. The DNA sequence of the AAVS1 binding site is shown and the location of TALEN Left and TALEN Right are underlined. (b) Graphical representation of data for the GFP-AAVS1 SplitAx with the TALEN Left and TALEN Right. Cells not transfected with a plasmid (-). Data shown as +STDev (n = 3).
(DOCX)

S4 Fig Targeting the C-Terminus of the SOX6 locus using hCAS9 and SOX 6 specific gRNA in human iPS cells. (a) Schematic diagram of the SOX6 targeting vector consisting of Left Homology Arm, T2A peptide, Blue Fluorescent Protein (BFP), Poly A (PA), Lox P sites (black triangles), EF1 alpha promoter, mOrange and Right Homology Arm (not to scale). (b) Schematic illustration of the SOX6 locus and exon 16 at the target site between the Left Homology, Right Homology Arm. (c) Targeted SOX6 locus with the SOX6 targeting vector. Arrows indicate primers used to screen 3’ end of the targeting site and solid bars indicate the PCR amplicons. (d) PCR products from 3’ PCR using primers P1 and P2. Clones 1, 2, 3 and 4, whilst Vec is the vector backbone and 0 is the negative control. (e) PCR products from 3’ PCR using primers P1 and P3. Clones 1, 2, 3, and 4 whilst Vec is the vector backbone and 0 is the negative control. Lanes 1 and 4 are positive for the targeting event but appear to have a different size PCR amplicon. This may be the result of chew back during cloning. (f) Sequencing trace clone 1 of the 3’ external PCR showing that this PCR amplicon is specific to the SOX6 locus.
(DOCX)

S5 Fig Comparison of the amino acid sequence between GFP and ZsGreen1. The critical residue at position 158 where the genome editing binding site is inserted is highlighted.
(DOCX)

S6 Fig Targeting the C-Terminus of the *Cfms* locus using the D10A nickase and Cfms-guide RNAs. (a) Schematic diagram of the Cfms targeting vector consisting of Left Homology Arm, T2A peptide, Matrix Metaloproteinase 12 cDNA (MMP12), Poly A (PA), Lox P sites (black triangles), PGK promoter and neomycin transferase gene (not to scale). (b) Schematic illustration of the Cfms locus and exon 9 at the target site between the Left Homology, Right Homology Arm. (c) Targeted Cfms locus with the Cfms targeting vector. Arrows indicate primers used to screen 3’ end of the targeting site and solid bars indicate the PCR amplicons. (d) PCR products from 3’ PCR using primers P1 and P2. Clones 1–8, whilst 0 is the negative control and Vec is the vector backbone. (e) PCR products from 3’ PCR using primers P1 and P3. Clones 1–8, whilst 0 is the negative control and Vec is the vector backbone.
(DOCX)

S7 Fig DNA sequence of retored open reading frames. Examples of sequences repaired by non-homologous end joining resulting in mutations that restore the open reading frame.
(DOCX)

S1 Table List of primers. Sequence of all primers used in this study.
(DOCX)
