# Supplementary material for: SplitAx: A novel method to assess the function of engineered nucleases
Source: PLoS One. 2017 Feb 17;12(2):e0171698. doi: 10.1371/journal.pone.0171698 (PMC5315338; doi:10.1371/journal.pone.0171698)
Supplement: S1 Fig — (a)DNA and translated protein sequences of GFP. Amino acid Q158 is marked in red. This is the position in which the genome editing binding site is inserted to split the GFP-N-terminus from the GFP-C-terminus. (b)DNA sequence of AAVS1-GFP-SplitAx vector.Amino acid Position 158 is marked in red, followed by Not1 restriction sites (underlined). AAVS1 genome editing binding site (shaded yellow) followed by Xho1 restriction sites (broken line). (c)DNA and translated protein sequence of AAVS1-GFP-SplitAx vector. Amino acid position 158 is shown in red and stop codons shown as dashes (-). (d)DNA and translated protein sequence of AAVS1-GFP-SplitAx following genome editing. Following a 1 bp deletion at nucleotide position 519 the complete GFP open reading frame is restored. (DOCX) [file pone.0171698.s001.docx]

**S1 Fig. DNA and translated protein sequences of GFP and AAVS1-GFP-SplitAx vector.**

(a)DNA and translated protein sequences of GFP. Amino acid Q158 is marked in red. This is the position in which the genome editing binding site is inserted to split the GFP-N-terminus from the GFP-C-terminus.

atggtgagcaagggcgaggagctgttcaccggggtggtgcccatcctggtcgagctggac

M V S K G E E L F T G V V P I L V E L D

ggcgacgtaaacggccacaagttcagcgtgtccggcgagggcgagggcgatgccacctac

G D V N G H K F S V S G E G E G D A T Y

ggcaagctgaccctgaagttcatctgcaccaccggcaagctgcccgtgccctggcccacc

G K L T L K F I C T T G K L P V P W P T

ctcgtgaccaccctgacctacggcgtgcagtgcttcagccgctaccccgaccacatgaag

L V T T L T Y G V Q C F S R Y P D H M K

cagcacgacttcttcaagtccgccatgcccgaaggctacgtccaggagcgcaccatcttc

Q H D F F K S A M P E G Y V Q E R T I F

ttcaaggacgacggcaactacaagacccgcgccgaggtgaagttcgagggcgacaccctg

F K D D G N Y K T R A E V K F E G D T L

gtgaaccgcatcgagctgaagggcatcgacttcaaggaggacggcaacatcctggggcac

V N R I E L K G I D F K E D G N I L G H

aagctggagtacaactacaacagccacaacgtctatatcatggccgacaagcagaagaac

K L E Y N Y N S H N V Y I M A D K Q K N

ggcatcaaggtgaacttcaagatccgccacaacatcgaggacggcagcgtgcagctcgcc

G I K V N F K I R H N I E D G S V Q L A

gaccactaccagcagaacacccccatcggcgacggccccgtgctgctgcccgacaaccac

D H Y Q Q N T P I G D G P V L L P D N H

tacctgagcacccagtccgccctgagcaaagaccccaacgagaagcgcgatcacatggtc

Y L S T Q S A L S K D P N E K R D H M V

ctgctggagttcgtgaccgccgccgggatcactctcggcatggacgagctgtacaagtaa

L L E F V T A A G I T L G M D E L Y K -

(b) DNA sequence of AAVS1-GFP-SplitAx vector. Amino acid Position 158 is marked in red, followed by Not1 restriction sites (underlined). AAVS1 genome editing binding site (shaded yellow) followed by Xho1 restriction sites (broken line).

ATGGTGAGCAAGGGCGAGGAGCTGTTCACCGGGGTGGTGCCCATCCTGGTCGAGCTGGACGGCGACGTAAACGGCCACAAGTTCAGCGTGTCCGGCGAGGGCGAGGGCGATGCCACCTACGGCAAGCTGACCCTGAAGTTCATCTGCACCACCGGCAAGCTGCCCGTGCCCTGGCCCACCCTCGTGACCACCCTGACCTACGGCGTGCAGTGCTTCAGCCGCTACCCCGACCACATGAAGCAGCACGACTTCTTCAAGTCCGCCATGCCCGAAGGCTACGTCCAGGAGCGCACCATCTTCTTCAAGGACGACGGCAACTACAAGACCCGCGCCGAGGTGAAGTTCGAGGGCGACACCCTGGTGAACCGCATCGAGCTGAAGGGCATCGACTTCAAGGAGGACGGCAACATCCTGGGGCACAAGCTGGAGTACAACTACAACAGCCACAACGTCTATATCATGGCCGACAAGCAGGCGGCCGCAAGCTTATCTGTCCCCTCCACCCCACAGTGGGGCCACTAGGGACAGGATTGGTGACAGAAAAGCCCCATCCTTGGATCCCTCGAGAAAGAACGGCATCAAGGTGAACTTCAAGATCCGCCACAACATCGAGGACGGCAGCGTGCAGCTCGCCGACCACTACCAGCAGAACACCCCCATCGGCGACGGCCCCGTGCTGCTGCCCGACAACCACTACCTGAGCACCCAGTCCGCCCTGAGCAAAGACCCCAACGAGAAGCGCGATCACATGGTCCTGCTGGAGTTCGTGACCGCCGCCGGGATCACTCTCGGCATGGACGAGCTGTACAAGTAA

(c) DNA and translated protein sequence of AAVS1-GFP-SplitAx vector. Amino acid position 158 is shown in red and stop codons shown as dashes (-).

atggtgagcaagggcgaggagctgttcaccggggtggtgcccatcctggtcgagctggac

M V S K G E E L F T G V V P I L V E L D

ggcgacgtaaacggccacaagttcagcgtgtccggcgagggcgagggcgatgccacctac

G D V N G H K F S V S G E G E G D A T Y

ggcaagctgaccctgaagttcatctgcaccaccggcaagctgcccgtgccctggcccacc

G K L T L K F I C T T G K L P V P W P T

ctcgtgaccaccctgacctacggcgtgcagtgcttcagccgctaccccgaccacatgaag

L V T T L T Y G V Q C F S R Y P D H M K

cagcacgacttcttcaagtccgccatgcccgaaggctacgtccaggagcgcaccatcttc

Q H D F F K S A M P E G Y V Q E R T I F

ttcaaggacgacggcaactacaagacccgcgccgaggtgaagttcgagggcgacaccctg

F K D D G N Y K T R A E V K F E G D T L

gtgaaccgcatcgagctgaagggcatcgacttcaaggaggacggcaacatcctggggcac

V N R I E L K G I D F K E D G N I L G H

aagctggagtacaactacaacagccacaacgtctatatcatggccgacaagcaggcggcc

K L E Y N Y N S H N V Y I M A D K Q A A

gcaagcttatctgtcccctccaccccacagtggggccactagggacaggattggtgacag

A S L S V P S T P Q W G H - G Q D W - Q

aaaagccccatccttggatccctcgagaaagaacggcatcaaggtgaacttcaagatccg

K S P I L G S L E K E R H Q G E L Q D P

ccacaacatcgaggacggcagcgtgcagctcgccgaccactaccagcagaacacccccat

P Q H R G R Q R A A R R P L P A E H P H

cggcgacggccccgtgctgctgcccgacaaccactacctgagcacccagtccgccctgag

R R R P R A A A R Q P L P E H P V R P E

caaagaccccaacgagaagcgcgatcacatggtcctgctggagttcgtgaccgccgccgg

Q R P Q R E A R S H G P A G V R D R R R

gatcactctcggcatggacgagctgtacaagtaa

D H S R H G R A V Q V

(d) DNA and translated protein sequence of AAVS1-GFP-SplitAx following genome editing. Following a 1 bp deletion at nucleotide position 519 the complete GFP open reading frame is restored.

atggtgagcaagggcgaggagctgttcaccggggtggtgcccatcctggtcgagctggac

M V S K G E E L F T G V V P I L V E L D

ggcgacgtaaacggccacaagttcagcgtgtccggcgagggcgagggcgatgccacctac

G D V N G H K F S V S G E G E G D A T Y

ggcaagctgaccctgaagttcatctgcaccaccggcaagctgcccgtgccctggcccacc

G K L T L K F I C T T G K L P V P W P T

ctcgtgaccaccctgacctacggcgtgcagtgcttcagccgctaccccgaccacatgaag

L V T T L T Y G V Q C F S R Y P D H M K

cagcacgacttcttcaagtccgccatgcccgaaggctacgtccaggagcgcaccatcttc

Q H D F F K S A M P E G Y V Q E R T I F

ttcaaggacgacggcaactacaagacccgcgccgaggtgaagttcgagggcgacaccctg

F K D D G N Y K T R A E V K F E G D T L

gtgaaccgcatcgagctgaagggcatcgacttcaaggaggacggcaacatcctggggcac

V N R I E L K G I D F K E D G N I L G H

aagctggagtacaactacaacagccacaacgtctatatcatggccgacaagcaggcggcc

K L E Y N Y N S H N V Y I M A D K Q A A

gcaagcttatctgtcccctccaccccacagtggggccatagggacaggattggtgacaga

A S L S V P S T P Q W G H R D R I G D R

aaagccccatccttggatccctcgagaaagaacggcatcaaggtgaacttcaagatccgc

K A P S L D P S R K N G I K V N F K I R

cacaacatcgaggacggcagcgtgcagctcgccgaccactaccagcagaacacccccatc

H N I E D G S V Q L A D H Y Q Q N T P I

ggcgacggccccgtgctgctgcccgacaaccactacctgagcacccagtccgccctgagc

G D G P V L L P D N H Y L S T Q S A L S

aaagaccccaacgagaagcgcgatcacatggtcctgctggagttcgtgaccgccgccggg

K D P N E K R D H M V L L E F V T A A G

atcactctcggcatggacgagctgtacaagtaa

I T L G M D E L Y K -
