# Supplementary material for: SplitAx: A novel method to assess the function of engineered nucleases
Source: PLoS One. 2017 Feb 17;12(2):e0171698. doi: 10.1371/journal.pone.0171698 (PMC5315338; doi:10.1371/journal.pone.0171698)
Supplement: S2 Fig — (a) Schematic diagram of the AAVS1-KLF1-mCherry reporter vector used to target the AAVS1 locus with zinc fingers P622L and P622R. Left Homology Arm, Splice Acceptor/2A peptide, Puromycin selectable cassette (P), Poly A (PA), KLF1 Promoter, mCherry reporter followed by the AAVS1 Right Homology Arm (not to scale). (b) Schematic illustration of the AAVS1 locus, endogenous promoter, exon 1 and the target site between the Left Homology, Right Homology Arm and exon 2. (c) Targeted AAVS1 locus with the KLF1 reporter vector. Arrows indicate primers used to screen 5’ and 3’ end of the targeting site and solid bars indicate the PCR amplicons. Screen for targeted events using 5’ primers A4 and A5 and 3’ primers are A1, A2 and A3. (d) PCR products from 5’ PCR using primers A4 and A5. Clone 9 indicates that it is targeted at the 5’ end. (e) PCR products from 3’ PCR using internal vector primers and an external primer. Clone 3 indicates a random targeting event whilst clone 9 indicates a targeted event into the AAVS1 locus. WT is genomic DNA from untreated iPS cells and 0 is a negative PCR control. (f) Sequencing trace clone 9 of the 3’ external PCR showing that this PCR amplicon is specific to the AAVS1 locus. (DOCX) [file pone.0171698.s002.docx]

**S2 Fig. Targeting of the AAVS1 locus with the KLF1 promoter mCherry reporter.**


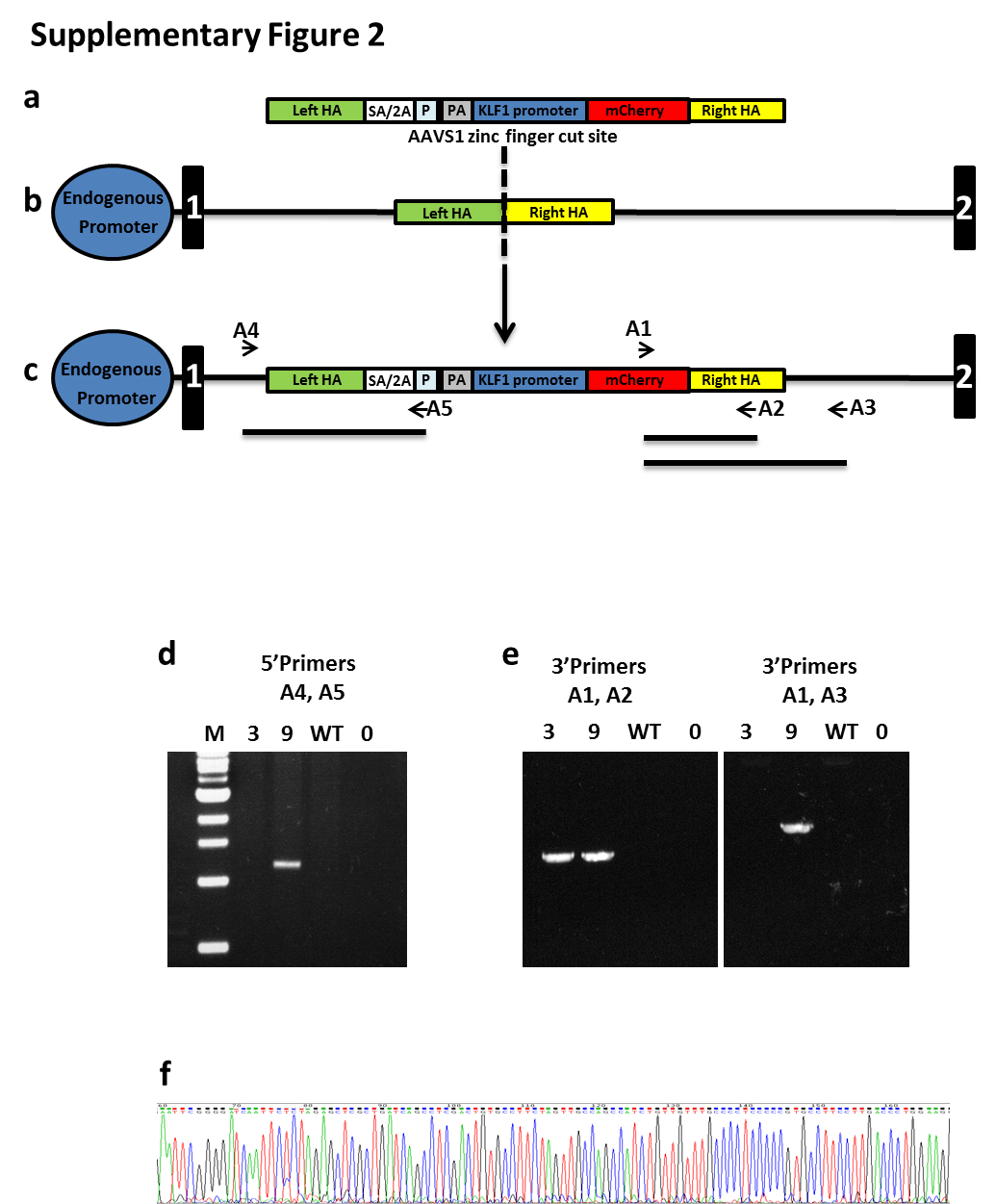


a) Schematic diagram of the AAVS1-KLF1-mCherry reporter vector used to target the *AAVS1* locus with zinc fingers P622L and P622R. Left Homology Arm, Splice Acceptor/2A peptide, Puromycin selectable cassette (P), Poly A (PA), KLF1 Promoter, mCherry reporter followed by the *AAVS1* Right Homology Arm (not to scale).

b) Schematic illustration of the *AAVS1* locus, endogenous promoter, exon 1 and the target site between the Left Homology, Right Homology Arm and exon 2.

c) Targeted *AAVS1* locus with the KLF1 reporter vector. Arrows indicate primers used to screen 5’ and 3’ end of the targeting site and solid bars indicate the PCR amplicons. Screen for targeted events using 5’ primers A4 and A5 and 3’ primers are A1, A2 and A3.

d) PCR products from 5’ PCR using primers A4 and A5. Clone 9 indicates that it is targeted at the 5’ end.

e) PCR products from 3’ PCR using internal vector primers and an external primer. Clone 3 indicates a random targeting event whilst clone 9 indicates a targeted event into the AAVS1 locus. WT is genomic DNA from untreated iPS cells and 0 is a negative PCR control.

f) Sequencing trace clone 9 of the 3’ external PCR showing that this PCR amplicon is specific to the *AAVS1* locus.
