# Supplementary material for: SplitAx: A novel method to assess the function of engineered nucleases
Source: PLoS One. 2017 Feb 17;12(2):e0171698. doi: 10.1371/journal.pone.0171698 (PMC5315338; doi:10.1371/journal.pone.0171698)
Supplement: S3 Fig — (a) Schematic of the GFP cDNA with the N-terminus and C-terminus separated by the AAVS1 binding site. The DNA sequence of the AAVS1 binding site is shown and the location of TALEN Left and TALEN Right are underlined. (b) Graphical representation of data for the GFP-AAVS1 SplitAx with the TALEN Left and TALEN Right. Cells not transfected with a plasmid (-). Data shown as +STDev (n = 3). (DOCX) [file pone.0171698.s003.docx]

**S3 Fig. Functional validation of the GFP-AAVS1 SplitAx reporter assay with AAVS1 TALENs.**


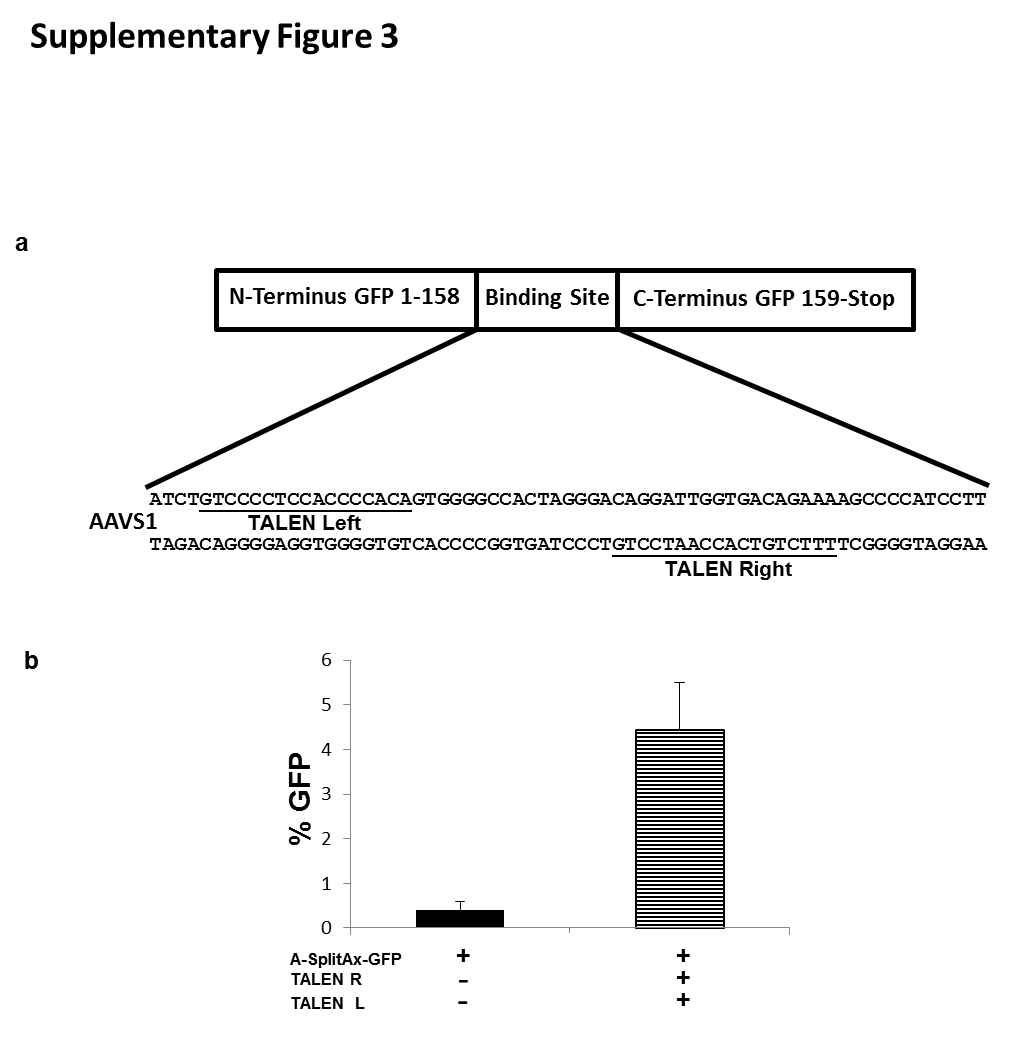


a) Schematic of the GFP cDNA with the N-terminus and C-terminus separated by the AAVS1 binding site. The DNA sequence of the AAVS1 binding site is shown and the location of TALEN Left and TALEN Right are underlined.

b) Graphical representation of data for the GFP-AAVS1 SplitAx with the TALEN Left and TALEN Right. Cells not transfected with a plasmid (-). Data shown as +STDev (n=3).
