# Supplementary material for: SplitAx: A novel method to assess the function of engineered nucleases
Source: PLoS One. 2017 Feb 17;12(2):e0171698. doi: 10.1371/journal.pone.0171698 (PMC5315338; doi:10.1371/journal.pone.0171698)
Supplement: S4 Fig — (a) Schematic diagram of the SOX6 targeting vector consisting of Left Homology Arm, T2A peptide, Blue Fluorescent Protein (BFP), Poly A (PA), Lox P sites (black triangles), EF1 alpha promoter, mOrange and Right Homology Arm (not to scale). (b) Schematic illustration of the SOX6 locus and exon 16 at the target site between the Left Homology, Right Homology Arm. (c) Targeted SOX6 locus with the SOX6 targeting vector. Arrows indicate primers used to screen 3’ end of the targeting site and solid bars indicate the PCR amplicons. (d) PCR products from 3’ PCR using primers P1 and P2. Clones 1, 2, 3 and 4, whilst Vec is the vector backbone and 0 is the negative control. (e) PCR products from 3’ PCR using primers P1 and P3. Clones 1, 2, 3, and 4 whilst Vec is the vector backbone and 0 is the negative control. Lanes 1 and 4 are positive for the targeting event but appear to have a different size PCR amplicon. This may be the result of chew back during cloning. (f) Sequencing trace clone 1 of the 3’ external PCR showing that this PCR amplicon is specific to the SOX6 locus. (DOCX) [file pone.0171698.s004.docx]

**S4 Fig. Targeting the C-Terminus of the SOX6 locus using hCAS9 and SOX 6 specific gRNA in human iPS cells.**


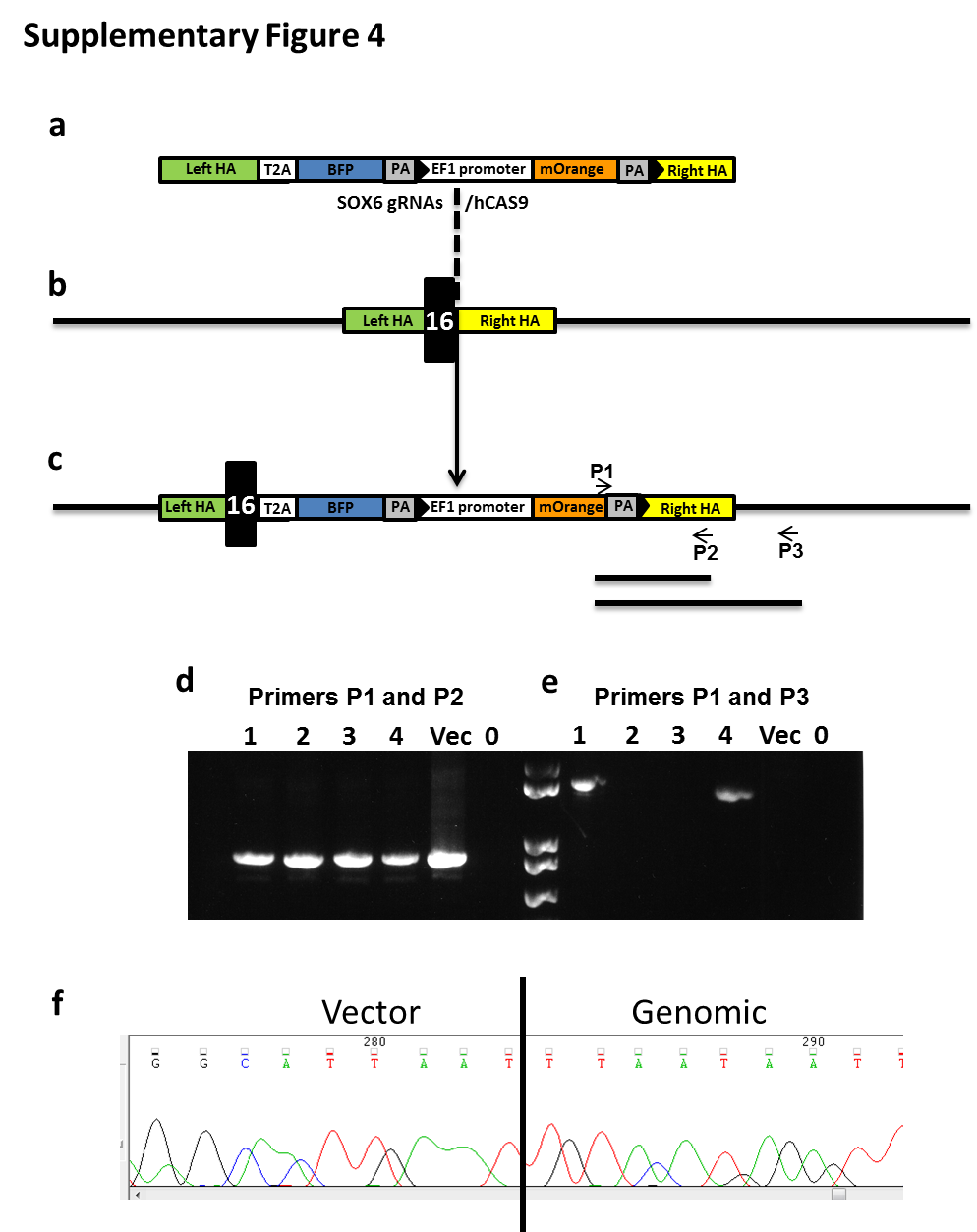


a) Schematic diagram of the SOX6 targeting vector consisting of Left Homology Arm, T2A peptide, Blue Fluorescent Protein (BFP), Poly A (PA), Lox P sites (black triangles), EF1 alpha promoter, mOrange and Right Homology Arm (not to scale).

b) Schematic illustration of the SOX6 locus and exon 16 at the target site between the Left Homology, Right Homology Arm.

c) Targeted SOX6 locus with the SOX6 targeting vector. Arrows indicate primers used to screen 3’ end of the targeting site and solid bars indicate the PCR amplicons.

d) PCR products from 3’ PCR using primers P1 and P2. Clones 1, 2, 3 and 4, whilst Vec is the vector backbone and 0 is the negative control.

e) PCR products from 3’ PCR using primers P1 and P3. Clones 1, 2, 3, and 4 whilst Vec is the vector backbone and 0 is the negative control. Lanes 1 and 4 are positive for the targeting event but appear to have a different size PCR amplicon. This may be the result of chew back during cloning.

f) Sequencing trace clone 1 of the 3’ external PCR showing that this PCR amplicon is specific to the SOX6 locus.
