# Supplementary material for: SplitAx: A novel method to assess the function of engineered nucleases
Source: PLoS One. 2017 Feb 17;12(2):e0171698. doi: 10.1371/journal.pone.0171698 (PMC5315338; doi:10.1371/journal.pone.0171698)

**S5 Fig. Comparison of the amino acid sequence between GFP and ZsGreen1.**

The critical residue at position 158 where the genome editing binding site is inserted is highlighted.


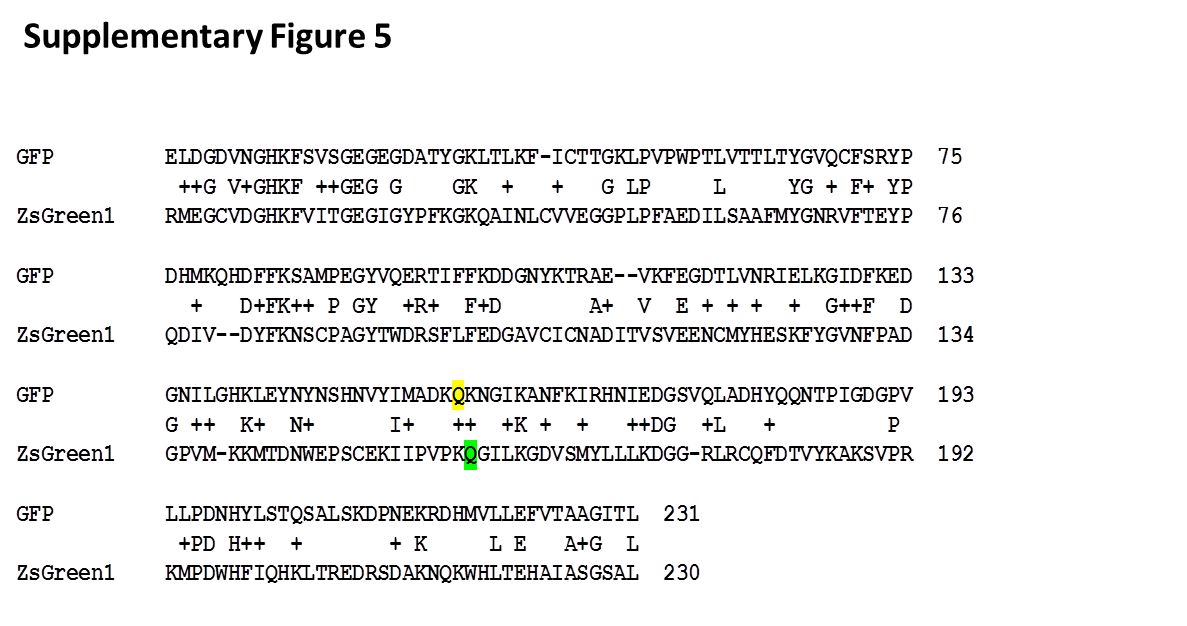

Supplement: S5 Fig — The critical residue at position 158 where the genome editing binding site is inserted is highlighted. (DOCX) [file pone.0171698.s005.docx]
