# Supplementary material for: SplitAx: A novel method to assess the function of engineered nucleases
Source: PLoS One. 2017 Feb 17;12(2):e0171698. doi: 10.1371/journal.pone.0171698 (PMC5315338; doi:10.1371/journal.pone.0171698)
Supplement: S6 Fig — (a) Schematic diagram of the Cfms targeting vector consisting of Left Homology Arm, T2A peptide, Matrix Metaloproteinase 12 cDNA (MMP12), Poly A (PA), Lox P sites (black triangles), PGK promoter and neomycin transferase gene (not to scale). (b) Schematic illustration of the Cfms locus and exon 9 at the target site between the Left Homology, Right Homology Arm. (c) Targeted Cfms locus with the Cfms targeting vector. Arrows indicate primers used to screen 3’ end of the targeting site and solid bars indicate the PCR amplicons. (d) PCR products from 3’ PCR using primers P1 and P2. Clones 1–8, whilst 0 is the negative control and Vec is the vector backbone. (e) PCR products from 3’ PCR using primers P1 and P3. Clones 1–8, whilst 0 is the negative control and Vec is the vector backbone. (DOCX) [file pone.0171698.s006.docx]

**S6 Fig. Targeting the C-Terminus of the *Cfms* locus using the D10A nickase and Cfms-guide RNAs.**

**
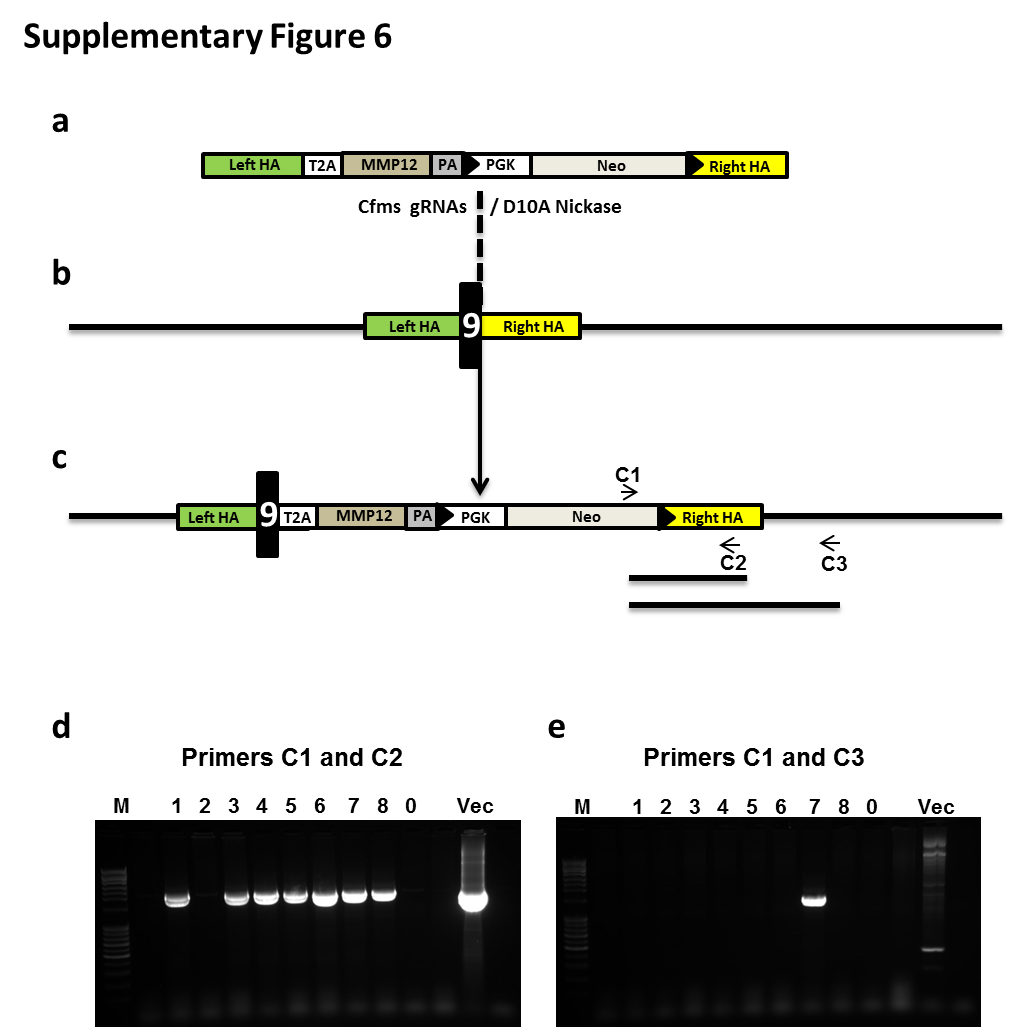
**

a) Schematic diagram of the Cfms targeting vector consisting of Left Homology Arm, T2A peptide, Matrix Metaloproteinase 12 cDNA (MMP12), Poly A (PA), Lox P sites (black triangles), PGK promoter and neomycin transferase gene (not to scale).

b) Schematic illustration of the Cfms locus and exon 9 at the target site between the Left Homology, Right Homology Arm.

c) Targeted Cfms locus with the Cfms targeting vector. Arrows indicate primers used to screen 3’ end of the targeting site and solid bars indicate the PCR amplicons.

d) PCR products from 3’ PCR using primers P1 and P2. Clones 1-8, whilst 0 is the negative control and Vec is the vector backbone.

e) PCR products from 3’ PCR using primers P1 and P3. Clones 1-8, whilst 0 is the negative control and Vec is the vector backbone.
