# Supplementary material for: SplitAx: A novel method to assess the function of engineered nucleases
Source: PLoS One. 2017 Feb 17;12(2):e0171698. doi: 10.1371/journal.pone.0171698 (PMC5315338; doi:10.1371/journal.pone.0171698)
Supplement: S7 Fig — Examples of sequences repaired by non-homologous end joining resulting in mutations that restore the open reading frame. (DOCX) [file pone.0171698.s007.docx]

**S7 Fig.**

Examples of sequences repaired by NHEJ resulting in mutations that restore the open reading frame.

**Δ10**

GTCTATATCATGGCCGACAAGCAGGCGGCCGCAAGCTTATCTGTCCCCTCCACCCCACAGTGGGGCCACTAGGGA

GTCTATATCATGGCCGACAAGCAGGCGGCCGCAAGCTTATCTGTCCCCTCCACCCCACAGT----------GGGA

CAGGATTGGTGACAGAAAAGCCCCATCCTTGGATCCCTCGAGAAAGAACGGCATCAAGGTGAACTTCAAGATCCG

CAGGATTGGTGACAGAAAAGCCCCATCCTTGGATCCCTCGAGAAAGAACGGCATCAAGGTGAACTTCAAGATCCG

**Δ79 +53,** GTCTATATCATGGCCGACAAGCAGGCGGCCGCAAGCTTATCTGTCCCCTCCACCCCACAGTGGGGCCACTAGGGA

GTCTATATCATGGCCGACAAGCAGGCGGCCGCGTCACCTGCAGCTGCCCAGACCTGGCACCCAGGAGAGGAGCAG

CAGGATTGGTGACAGAAAAGCCCCATCCTTGGATCCCTCGAGAAAGAACGGCATCAAGGTGAACTTCAAGATCCG

GCAGGGTCAG--------------------------CTCGAGAAAGAACGGCATCAAGGTGAACTTCAAGATCCG

**Δ91 +110** GTCTATATCATGGCCGACAAGCAGGCGGCCGCAAGCTTATCTGTCCCCTCCACCCCACAGTGGGGCCACTAGGGA

GTCTATATCATGGCCGACAAGCAGCGCCTCATAGCCTCATGGACACCTGTGTCGCCACCTTCCATGTGAAGCAGC

CAGGATTGGTGACAGAAAAGCCCCATCCTTGGATCCCTCGAG-------------------AAAGAACGGCATCA

AAGGCCCAGGTCCCGAAAGATGCAGTGACTTTTTGTCGTGGCAGCCAGTGGTGACTGGATTAAAGAACGGCATCA

**Δ93 +83,**

GTCTATATCATGGCCGACAAGCAGGCGGCCGCAAGCTTATCTGTCCCCTCCACCCCACAGTGGGGCCACTAGGGA

GTCTATATCATGGCCGACAAGCAGGACATTTCACCCCCCTCCCCAACAAAGAGTTATTAAAGAGCCCGCATGCAT

CAGGATTGGTGACAGAAAAGCCCCATCCTTGGATCCCTCGAGAAAGAACGGCATCAAGGTGAACTTCAAGATCCG

TTGTGGCTCCACAATTACATCAGCAGAATGGT-----------AAGAACGGCATCAAGGTGAACTTCAAGATCCG
