# Supplementary material for: SplitAx: A novel method to assess the function of engineered nucleases
Source: PLoS One. 2017 Feb 17;12(2):e0171698. doi: 10.1371/journal.pone.0171698 (PMC5315338; doi:10.1371/journal.pone.0171698)
Supplement: S1 Table — Sequence of all primers used in this study. (DOCX) [file pone.0171698.s008.docx]

**S1 Table. List of primers**

Sequence of all primers used in this study

| **Primer name/gene** | **Sequence** |
| --- | --- |
| KLF1a forward | GGTACCCTCAGCCTCCTGAGTAGCTGGGACTA |
| KLF1a reverse | GGTACC GTCCTGCGCCTCTTCGGAGCGCCA |
| mCherry forward | GGTACCATGGTGAGCAAGGGCGAGGAGGATAAC |
| mCherry rev | GAATTC TTACTTGTACAGCTCGTCCATGCCGC |
| βGlobin PA forward | GAATTC GGGGATCAATTCTCTAGAGCTCGCTGATCAGC |
| βGlobin PA reverse | GAATTCTAAGGGTTCCGCAAGCTCTAGTCGAGCCCCA |
| KLF1PZpuro forward | ACCGGTCTCATGTTGGGCAGAATCAAGGAGCA |
| PZdage reverse | ACCGGTTAAGGGTTCCGCAAGCTCTAGTCGAGCCCCA |
| A1 Cherry 5 forward | CGAGGACTACACCATCGTGGAAC |
| A2 AAVS1Iint_reverse | TCTCCTGGGCTTGCCAAGGACTCAAAC |
| A3 AAVS1ext reverse | CACACCCACACCTGACCCAAACCCAG |
| A4 5’external for | CGGAACTCTGCCCTCTAACGCTGCCG |
| A5 PZpuro reverse | TGAGGAAGAGTTCTTGCAGCT |
| S1 5’ SOX6Homforward | GCGGCCGCGTGAGGCAGCGTATCTGGTGTCTAC |
| S2 5’SOX6Hom reverse | GCGGCCGCAGTTGGCACTGACAGCCTCCGGGGCTTC |
| S3 3’SOX6Hom forward | CTCGAGAATTGTTTCGTAAAGTGTGAGAC |
| S4 3’SOX6Hom reverse | CTCGAGAATTAATGCCACCAACAATTGAA |
| E1 EF1-forward | GAATTCATAACTTCGTATAATGTATGCTATACGAAGTTATGTAATTCATAC  AAAAGGCTCGCCCCTG |
| E2 EF1 reverse | GAATTCGATATCTCACGACACCTGAAATGGAAGAAAAAAACT |
| P1 mOrange_forward | GACATCACCTCCCACAACGAGGACTACACC |
| P2 3’SOX6INT reverse | TCACAATATGAAATCCTCAGTTGT |
| P3 3’SOX6EXT reverse | TAAGTGTAAGACTATATACCTATATC |
| C4 Cfms 5'Hom forward | GGTACCCAAGACAGAGCAAGGGCTGGCA |
| C5 Cfms 5'Hom reverse | GGTACCGCAGAACTGGTAGTTGTTA |
| C6 Cfms 3'Home forward | GCGGCCGCAGTGGGAGGGAGAGCCGAG |
| C7 Cfms 3'Hom reverse | GCGGCCGCCTGGCTGTGTTAATGCTG |
| C1 Cfms_forward | GCTTCTGAGGCGGAAAGAACCAG |
| C2 Cfms reverse | AAGCTGAGGGCGAAGTCCATATG |
| C3 Cfms reverse | CAAACAGCAGCATGGGCTCAGGT |
